# The extent of the raphe in bicuspid aortic valves is associated with aortic regurgitation and aortic root dilatation

**DOI:** 10.1007/s12471-015-0784-4

**Published:** 2016-01-12

**Authors:** W. M. C. Koenraadt, N. Grewal, O. Y. Gaidoukevitch, M. C. DeRuiter, A. C. Gittenberger-de Groot, M. M. Bartelings, E. R. Holman, R. J. M. Klautz, M. J. Schalij, M. R. M. Jongbloed

**Affiliations:** Department of Cardiology, Leiden University Medical Center, Albinusdreef 2, 2333 ZA Leiden, The Netherlands; Department of Cardiothoracic Surgery, Leiden University Medical Center, Leiden, The Netherlands; Department of Anatomy & Embryology, Leiden University Medical Center, PO Box 9600, 2300 RC Leiden, The Netherlands

**Keywords:** Aortic diseases, Echocardiography, Heart defects, Congenital, Valvular heart disease

## Abstract

**Background:**

The clinical course of bicuspid aortic valves (BAVs) is variable. Data on predictors of aortopathy and valvular dysfunction mainly focus on valve morphology.

**Aim:**

To determine whether the presence and extent of the raphe (fusion site of valve leaflets) is associated with the degree of aortopathy and valvular dysfunction in patients with isolated BAV and associated aortic coarctation (CoA).

**Methods:**

Valve morphology and aortic dimensions of 255 BAV patients were evaluated retrospectively by echocardiography.

**Results:**

BAVs with a complete raphe had a significantly higher prevalence of valve dysfunction (especially aortic regurgitation) than BAVs with incomplete raphes (82.9 vs. 66.7 %, *p* = 0.01). Type 1A BAVs (fusion of right and left coronary leaflets) and complete raphe had larger aortic sinus diameters compared with the rest of the population (37.74 vs. 36.01, *p* = 0.031). Patients with CoA and type 1A BAV had significantly less valve regurgitation (13.6 vs. 55.8 %, *p* < 0.001) and smaller diameters of the ascending aorta (33.7 vs. 37.8 mm, *p* < 0.001) and aortic arch (25.8 vs. 30.2 mm, *p* < 0.001) than patients with isolated BAV.

**Conclusions:**

Type 1A BAV with complete raphe is associated with more aortic regurgitation and root dilatation. The majority of CoA patients have incomplete raphes, associated with smaller aortic root diameters and less valve regurgitation.

## Introduction

Bicuspid aortic valve (BAV) is the most common congenital cardiac malformation, with a clear male predominance and an estimated prevalence of 0.5− 2 % [1–3]. BAV can occur in an isolated form or in association with other congenital malformations, such as coarctation of the aorta (CoA). The prevalence of BAV in CoA patients is reported to be as high as 60 % [4, 5].

Although some patients with isolated BAV remain asymptomatic throughout their lifetime, others develop severe cardiac complications from an early age onwards, such as aortic valve stenosis, aortic insufficiency and/or endocarditis. However, the first presentation can also be a clinically relevant aortic wall abnormality, including ascending aortic dilatation (reported to occur in 45 % of patients [6]) or rupture of the ascending aorta. Identifying patients who are prone to develop complications is a major challenge [7].

**Fig. 1:**
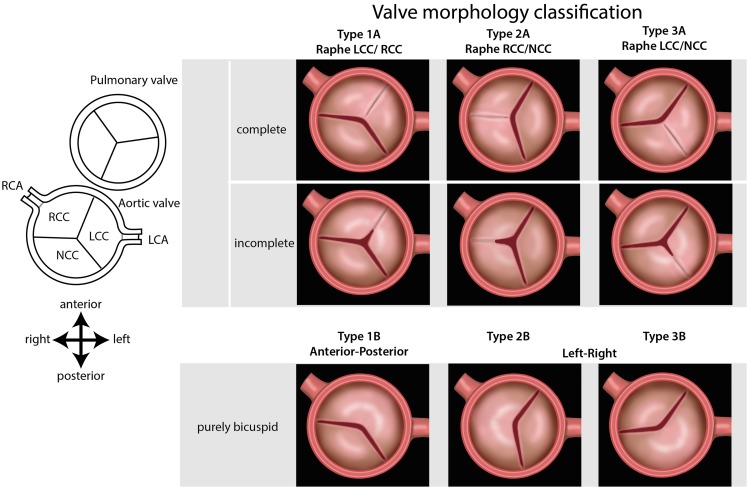
Schematic overview of bicuspid aortic valve (BAV) morphologies. The drawings are oriented in an echocardiographic view of the aortic valve. *Upper panel*: The three major valve morphologies observed in BAV patients are described as three different types based on the valve leaflet orientation: type 1,2 and 3. The extent of the raphe is indicated as analysed in this study (incomplete or complete). *Lower panel*: Strictly bicuspid valves (without a raphe) are defined as a subgroup B. Modified after Schaefer et al. [9]

It is now recognised that BAV should not be considered to be one single entity, but that distinct morphological phenotypes are distinguishable based on the presence and number of raphes, as classified by Sievers et al. [8]. Most BAVs consist of one free leaflet and two leaflets that are conjoined (or have failed to separate during embryonic development). The term ‘raphe’ defines the conjoined area of the two underdeveloped leaflets turning into a malformed commissure between both leaflets [8]. When no raphe is present, the valve is called strictly bicuspid. Schaefer et al. identified three BAV morphologies: type 1 with fusion of the right and left coronary cusp; type 2 with right and non-coronary cusp fusion; and the rare type 3 with left and non-coronary cusp fusion [9]. In this study, this classification was followed, with addition of A (for valves with a raphe) or B (for strictly bicuspid valves, Fig. 1).

Several studies have shown before that different BAV phenotypes have a different clinical outcome, regarding valvular dysfunction and aortopathy [9–14]. To our knowledge, however, the extent of the raphe (i.e. the extent to which the leaflets are conjoined) has never been taken into account. Therefore, the purpose of this study was to determine whether the extent of the raphe is associated with the degree of aortopathy and valvular dysfunction.

As stated before, the most common associated congenital malformation of BAV is coarctation of the aorta (CoA). Whereas in isolated BAV the most common morphology is a fusion of the right and left coronary cusp (type 1A in 80 % of patients [9]), data on the distribution of BAV morphology in CoA patients are limited [15]. Therefore, a sub-analysis was performed in patients with BAV and CoA to see if there was a difference in BAV morphology and extent of the raphe.

## Methods

### Study population

All patients who had undergone transthoracic echocardiography (TTE) between 2005 and 2010 and had been diagnosed with BAV disease were identified from the echocardiography database. A total of 263 BAV patients were selected, in 8 patients the diagnosis of BAV was not correct [16], thus 255 patients remained. Clinical and echocardiographic data were collected and analysed for these patients from the departmental Cardiology Information System (EPD-Vision®, LUMC) and the echocardiography database, respectively. For this analysis of clinically acquired data, the Institutional Review Board waived the need for patient written informed consent.

### Classification of valve morphology

As from a developmental point of view a morphological spectrum may exist in which the extent of the raphe can be regarded as a continuum, in the current study we chose to describe the major valve morphologies observed in BAV patients as 3 different types (type 1A, 2A and 3A, based on valve leaflet orientation, modified after Schaefer et al. [9]), in which the extent of the raphe can vary (Fig. 1). We defined fusion of the right and left coronary cusp as type 1A BAV, fusion of the right and non-coronary cusp as type 2A BAV and fusion of the left and non-coronary cusp as type 3A BAV. Strictly bicuspid valves (i.e. a valves without a raphe) were defined as being either type 1, 2 or 3, based on the orientation of their leaflets, and referred as a subgroup B (Fig. 1).

### Echocardiography

**Fig. 2:**
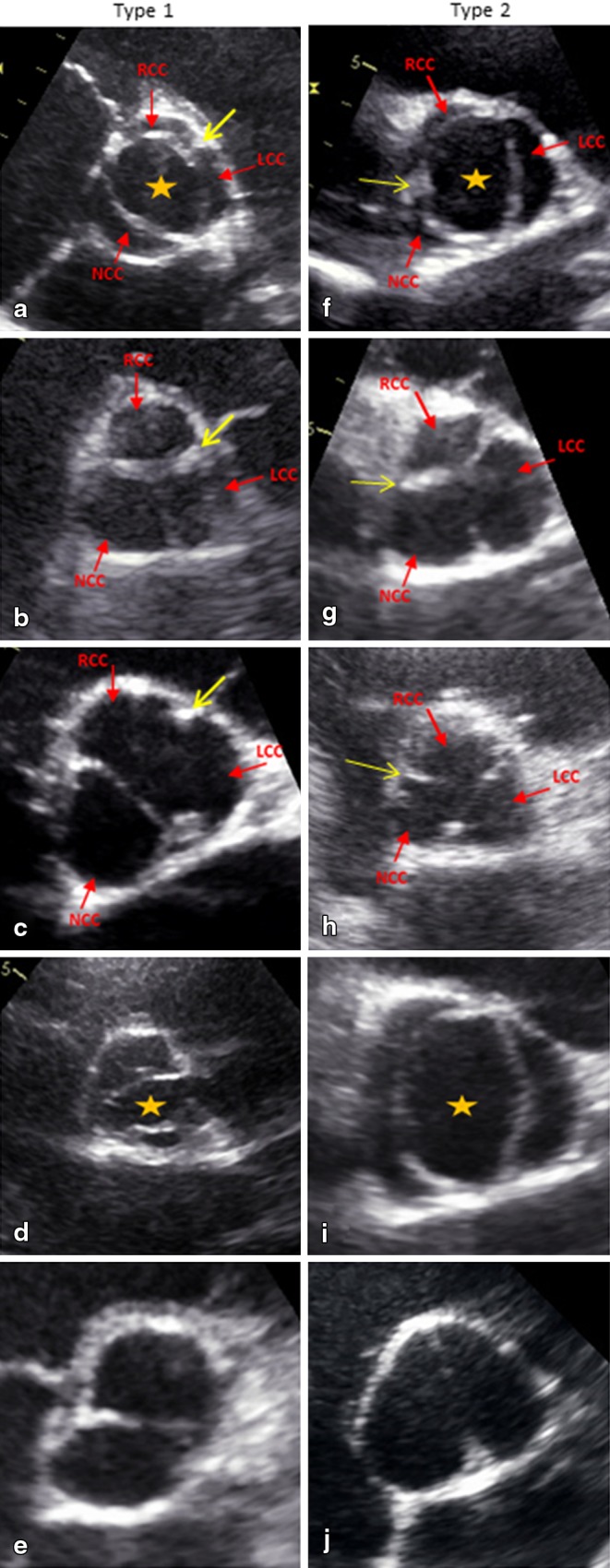
Representative examples of different bicuspid aortic valve (BAV) types as seen from the short-axis parasternal view on echocardiography. The *yellow arrow* indicates the raphe. The orange star indicates the typical fish mouth opening of the valve. **a** Type 1A in open position. **b** Type 1A with complete raphe in closed position. **c** Type 1A with incomplete raphe in closed position. **d** Type 1B in open position. **e** Type 1B in closed position. **f** Type 2A in open position. **g** Type 2A with complete raphe in closed position. **h** Type 2A with incomplete raphe in closed position. **i** Type 2B in open position. **j** Type 2B in closed position

Transthoracic echocardiography was performed using a GE Vivid7 or E9 (GE-Vingmed, Horten, Norway) ultrasound machine with standard views from the parasternal, subcostal, suprasternal and apical windows. The aortic valve was examined in the two-dimensional parasternal short-axis view and classified as bicuspid when two cusps could be clearly identified and the typical ‘fish-mouth opening’ of the valve was observed (Fig. 2). Diameters of the aortic root and ascending aorta were determined in the parasternal long-axis view.

### Echocardiographic analysis

All echocardiograms were evaluated by an experienced observer, using GE Medical System’s EchoPac, Version 7 (110.0.0, GE-Vingmed, Horten, Norway). The aortic valve was evaluated in a cross-sectional view for the presence and extent of a raphe. For valves where a raphe could be distinguished (subgroup A), distinction was made between a complete raphe and an incomplete raphe. Cases where no raphe was detected (subgroup B) were defined as strictly bicuspid valves. Diameters of aortic sinus, ascending aorta and aortic arch were measured from leading edge to leading edge in end-diastole according to the European Association of Echocardiography recommendations [17]. Aortic annular diameter was measured from inner edge to inner edge during systole. All measurements were in mm, rounded to 2 significant figures. The ascending aorta was considered dilated at a diameter of > 38 mm.

Valvular dysfunction was defined as aortic stenosis or regurgitation. European Association of Echocardiography (EAE) recommendations were used for determining severity of aortic stenosis and regurgitation, grading from mild to severe [18, 19]. Subgroup analysis was performed in patients with a history of CoA, the same protocol was followed in this group.

## Statistical analysis

All collected data were registered in a Microsoft Office Access 2003 database. The database was exported into IBM SPSS Statistics Version 20 for computing variables and statistical analysis. Independent samples T-tests were used to compare means of numerical data in two categories. One-way ANOVA tests were used for comparing numerical data in more than two categories. Cross-tabulations were made for binary categorical data, on which chi-square goodness-of-fit-tests were performed to test for independence. For sets of independent numerical data linear regression analysis was used to evaluate trends. Similarly, trends for binary categories were evaluated with binary logistic regression to correct for possible confounding factors such as age and gender. All statistical analyses were two-tailed and considered significant if *p* < 0.05.

## Results

### Patient characteristics

**Fig. 3:**
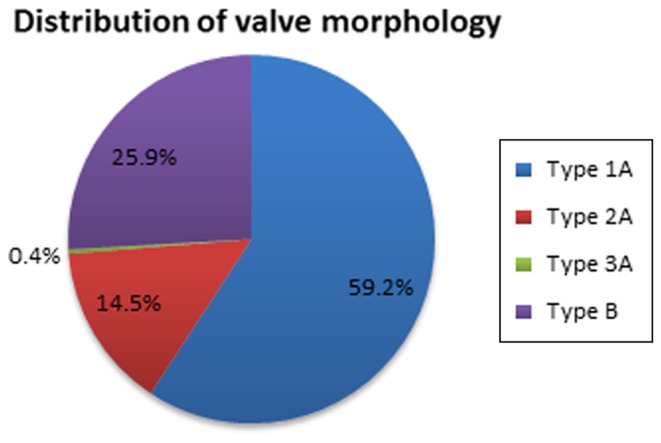
Schematic overview of the distribution of valve morphologies in the study population

A total of 255 patients with BAV (age 18–85 years, mean 48 ± 15 years) were identified and analysed [16]. Of these, 179 were male (70.2 %) and 76 female (29.8 %). Patient characteristics and echocardiographic data are summarised in Table 1. Baseline characteristics were not significantly different between the BAV subtypes (Table 2). The distribution of valve morphology is shown in Fig. 3.

**Table 1:** Patient characteristics

Variable	*n* (%)
Gender	
Male	179 (70.2)
Female	76 (29.8)
Bicuspid aortic valve morphology	
Type 1A	151 (59.2)
Type 2A	37 (14.5)
Type 3A	1 (0.4)
Strictly bicuspid (type B) Type 1B Type 2B	66 (25.9) 42 (63.6) 24 (36.4)
Extent of the raphe	
Complete	111 (43.5)
Incomplete	78 (30.6)
Valve surgery	66 (25.9)
Mitral valve replacement	1 (0.39)
Mitral valve repair	2 (0.78)
Aortic valve replacement	50 (19.6)
Aortic valve repair	10 (3.9)
Bentall	22 (8.6)
Tricuspid valve repair	1 (0.39)
Pulmonary valve replacement	1 (0.39)
Congenital defects	51 (20.0)
Aortic coarctation	39 (15.3)
Atrial septal defect	1 (0.39)
Ventricular septal defect	11 (4.3)
Marfan	2 (0.78)
Common truncus	1 (0.39)
Patent ductus arteriosus	5 (2.0)
Valvular dysfunction	189 (74.1)
Aortic valve stenosis	120 (47.1)
Aortic valve regurgitation	120 (47.1)
Mitral valve regurgitation	16 (6.3)
Tricuspid valve regurgitation	8 (3.1)
Pulmonary valve stenosis	1 (0.39)
Pulmonary valve regurgitation	1 (0.39)
Medication	140 (54.9)
ACE inhibitors	91 (35.7)
Statins	61 (23.9)
Carbasalate calcium	45 (17.6)
Oral anticoagulants	43 (16.9)
Antiarrhythmic drugs	33 (12.9)
Risk factors	117 (45.9)
Hypertension	65 (25.5)
Smoking	54 (21.2)
Diabetes	11 (4.3)
Cerebrovascular accident	14 (5.5)
Hypercholesterolaemia	33 (12.9)
Peripheral arterial disease	7 (2.7)

**Table 2:** Patient characteristics of different BAV subgroups

	Total*n* = 255	Type 1A *n* = 151	Type 2A *n* = 37	Type 3A *n* = 1	Type B *n* = 66	*p*-value
Gender (male)	179 (70 %)	110 (73 %)	23 (62 %)	0 (0 %)	46 (70 %)	0.260
Age (years)	48 ± 15	49 ± 14	46 ± 17	42 ± 25	47 ± 15	0.515
Risk factors						
Diabetes	11 (4 %)	8 (5 %)	2 (5 %)	0 (0 %)	1 (2 %)	0.609
Hypertension	65 (26 %)	44 (29 %)	8 (22 %)	0 (0 %)	13 (20 %)	0.414
Hypercholesterolaemia	33 (13 %)	23 (15 %)	3 (8 %)	0 (0 %)	7 (11 %)	0.585
Smoking	51 (20 %)	31 (21 %)	8 (22 %)	0 (0 %)	12 (18 %)	0.925
Medication						
Carbasalate calcium	45 (18 %)	19 (13 %)	8 (22 %)	1 (100 %)	17 (26 %)	0.013
Statins	59 (23 %)	31 (21 %)	9 (24 %)	1 (100 %)	18 (27 %)	0.207
ACE inhibitor	89 (35 %)	59 (39 %)	12 (32 %)	0 (0 %)	18 (28 %)	0.352
Oral anticoagulants	42 (17 %)	28 (19 %)	6 (16 %)	0 (0 %)	0 (0 %)	0.068

### More valvular dysfunction in BAVs with complete raphe

A total of 120 patients were diagnosed with aortic stenosis, the same number of patients showed aortic regurgitation (Table 1). Mean age was not significantly different in the different BAV subgroups (*p* = 0.515, Table 2) or in patients with incomplete and complete raphes (*p* = 0.357). There were no significant differences in valvular function between the different morphological subtypes. However, BAVs with a complete raphe had a significantly higher prevalence of valvular dysfunction than BAVs with an incomplete raphe (82.9 vs. 66.7 %, *p* = 0.01, Table 3). When specifying this for type of valvular dysfunction, this difference remained significant in patients with aortic regurgitation (56.8 vs. 42.3 %, *p* = 0.05), whereas in patients with aortic stenosis there was no difference between patients with a complete or incomplete raphe (49.5 vs. 43.6 %, *p* = 0.419, Table 3). In type 1A BAVs with a complete raphe the difference in prevalence of aortic regurgitation was even more outspoken compared with the rest of the study population (57.6 vs. 41.8 % *p* = 0.017).

**Table 3:** Valve morphology as related to dysfunction

Valve dysfunction	Complete raphe	Incomplete raphe	*p* value
Valve dysfunction (*n* = 144)	92 (82.9 %)	52 (66.7 %)	0.01
Aortic regurgitation (*n* = 96)	63 (56.8 %)	33 (42.3 %)	0.05
Aortic stenosis (*n* = 89)	55 (49.5 %)	34 (43.6 %)	NS

### Larger aortic diameter in type 1A BAVs and complete raphe

All type 1A BAVs, compared with type 2A BAVs, showed a significantly larger sinus (37.29 vs. 33.89 mm, *p* = 0.001, Table 4). Valves with a complete raphe had significantly larger aortic diameters at the level of the ascending aorta, as compared with valves with an incomplete raphe (38.02 vs. 35.01, *p* = 0.041). At the level of the aortic sinus and aortic arch this difference was not significant (Table 5). Type 1A BAVs and a complete raphe also showed a significant difference in sinus diameter (37.74 vs. 36.01, *p* = 0.031) compared with the rest of the study population.

**Table 4:** Valve morphology as related to aortic diameter; type 1A versus type 2A

Aortic diameter	Type 1A	Type 2A	*p* value
Sinus (mm)	37.29 ± 5.88	33.89 ± 4.84	0.001
Ascendens (mm)	37.34 ± 6.75	36.33 ± 7.03	NS
Arch (mm)	29.30 ± 5.45	30.94 ± 4.70	NS

### More incomplete raphes in CoA patients

Patients with CoA constituted the largest group of patients with associated congenital cardiac malformations (*n* = 39). Patients with a history of CoA were 9 years younger than those without (40.7 vs. 49.3 years old, *p* < 0.001) and there was no statistical difference in gender (*p* = 0.47).

Distribution of BAV morphology in patients with CoA was identical to the rest of the study population, the majority having a type 1A BAV (56.4 % in patients with vs. 59.7 % in patients without CoA, *p* = 0.321). However, in the majority (72.7 %) of CoA patients and type 1A BAV an incomplete raphe was seen as compared with only 38.8 % in the population without CoA (Table 6).

**Table 5:** Valve morphology as related to aortic diameter in all types BAV with complete raphe versus all types BAV with incomplete raphe

Aortic diameter	Complete raphe	Incomplete raphe	*p* value
Sinus (mm)	36.91 ± 5.82	36.13 ± 5.81	NS
Ascendens (mm)	38.02 ± 6.53	35.91 ± 7.00	0.041
Arch (mm)	30.07 ± 5.16	29.21 ± 5.66	NS

Patients with CoA and type 1A BAV had significantly less valve regurgitation (13.6 vs. 55.8 %, *p* < 0.001) and significantly smaller diameters of the ascending aorta (33.7 vs. 37.8 mm, *p* < 0.001) and aortic arch (25.8 vs. 30.2 mm, *p* < 0.001) than type 1A patients with isolated BAV (Table 6).

## Discussion

Key findings of this study are (1) Patients with type 1A BAV and a complete raphe show more aortic regurgitation and root dilatation as compared with the rest of the study population; (2) The majority of CoA patients have an incomplete raphe and smaller aortic root diameters and less valve regurgitation.

### Extent of the raphe as predictor of outcome

An association has been described between aortic stenosis and type 2 BAVs [12, 20]; however, in those studies no distinction was made between the presence or not of a raphe. In the current study, the same trend was seen, but this finding was not significant (*p* = 0.252). Also a relation has been shown between type 1A BAVs and aortic regurgitation [21], which is in line with the current study. The most important findings of the current study, however, were related to the *extent* of the raphe. A complete raphe predisposed for larger aortic diameters and more valve regurgitation. To our knowledge, the extent of a raphe in BAV disease has not been studied previously as a prognostic factor. The worse outcome observed in patients with a complete raphe is possibly due to the fact that BAVs with incomplete raphes have a more physiological, tricuspid-like opening and therefore function better. BAVs with complete raphes seem to have more unevenly sized leaflets and smaller openings which may predispose to valve dysfunction.

Type 1A BAVs have been related to aortic sinus dilatation -which is in line with the current study- and type 2A BAVs have been associated with dilatation of the ascending aorta [9, 11, 13]. However, none of these studies take into account the extent of the raphe. The current study showed a significant difference in ascending aorta diameter between BAVs with a complete versus incomplete raphe. Differences in dilation might therefore be explained by the extent of the raphe, e.g. due to altered flow, although this remains speculative at this point.

Patients with type 1A BAVs and a complete raphe showed significantly more regurgitation and root dilation as compared with the rest of the study population. Therefore, type 1A BAVs can be regarded as the valve orientation with the highest risk, which is in line with previous studies [11, 22, 23]. This indicates that type 1A BAVs that also have a complete raphe should even be monitored more closely for valve regurgitation and aortopathy.

### Effect of CoA on BAV morphology and outcome

Subgroup analysis of the CoA group revealed that these patients are on average 9 years younger than the rest of the study population, which may be explained by the fact that these patients usually show symptoms earlier and are often referred from the paediatric cardiologist as soon as they reach adulthood. The prevalence of BAV in CoA patients is an estimated 60 % [4, 5]. The majority of patients in the current study had type 1A BAV, which corresponds to reports in the literature [15]. CoA patients had smaller aortic root diameters and less valve regurgitation, which might be explained by the fact that less CoA patients had a BAV with a complete raphe. Another explanation for the smaller aortic root diameters could be the younger age of CoA patients. The prevalence of stenosis was similar in the CoA group compared with the rest of the study population. This in contrast to earlier research which found an association between CoA and valve dysfunction [5], although in that study the extent of raphe was not taken into account.

## Study limitations

This was a retrospective analysis of clinically obtained patient data derived from a single centre. A retrospective analysis is subjected to selection bias, as the investigator self-selects the cases. All patients were followed in a tertiary referral centre, which may have led to a selection bias of more severely affected patients. Both biases were minimised by including consecutive BAV patients who underwent echocardiography between 2005 and 2010.

## Conclusions and clinical implications

This study shows that the extent of the raphe is of clinical importance, as a complete raphe predisposes to more valvular dysfunction and aortopathy. Moreover, patients with type 1A BAV and a complete raphe show more aortic regurgitation and root dilatation as compared with the rest of the study population. This could indicate that this group of patients needs closer monitoring and more regular follow-up.

On the other hand, this study shows that patients with a bicuspid aortic valve and coarctation of the aorta have smaller aortic root diameters and less valve regurgitation, possibly due to the fact that in this group of patients the majority have an incomplete raphe.

**Table 6:** Characteristics of patients with type 1A bicuspid aortic valves (BAVs) with coarctation of the aorta (CoA) versus without CoA

Variable	CoA	No CoA	*p* value
Type 1A BAV, *n* (%)	22 (56.4 %)	129 (59.7 %)	NS
Raphe			0.003
Incomplete, *n* (%)	16 (72.7 %)	50 (38.8 %)	
Complete, *n* (%)	6 (27.3 %)	79 (61.2 %)	
Ascendens (mm)	33.3 ± 6.06	37.8 ± 6.78	< 0.001
Arch (mm)	25.5 ± 4.77	30.2 ± 4.98	< 0.001
Valve regurgitation	3 (13.6 %)	72 (55.8 %)	< 0.001
Valve stenosis	10 (45.5 %)	56 (43.4 %	NS
